# Clustering Analysis of Emotional Expression, Personality Traits, and Psychological Symptoms

**DOI:** 10.3390/brainsci16040353

**Published:** 2026-03-25

**Authors:** Lingping Meng, Mingzheng Li, Xiao Sun

**Affiliations:** 1 School of Mental Health and Psychological Sciences, Anhui Medical University, Hefei 230032, China; 2345011099@stu.ahmu.edu.cn; 2 Hefei Comprehensive National Science Center, Institute of Artificial Intelligence, Hefei 230088, China; 3 Institute of Advanced Technology, University of Science and Technology of China, Hefei 230094, China

**Keywords:** personality traits, emotion expression, facial expression recognition, k-means cluster analysis, mental health risks, deep learning, AffectNet, convolutional neural network

## Abstract

**Highlights:**

**What are the main findings?**

- Young adults showed the highest levels of depression and anxiety, while older adults exhibited lower psychological symptom levels; conscientiousness increased significantly with age and showed the strongest age-related effect.
- K-means clustering identified three distinct emotional expression patterns, with the anger/disgust/fear cluster showing the highest neuroticism, anxiety, and depression scores, and the poorest social functioning.

**What are the implications of the main findings?**

- Neuroticism is the strongest predictor of mental health risk (OR = 4.58), while extraversion and conscientiousness serve as protective factors, supporting personality-based mental health screening tools.
- These findings provide empirical evidence for age-differentiated mental health interventions and highlight the value of integrating personality assessments into risk prediction models.

**Abstract:**

Background: This study examined age-related differences and interrelationships among psychological symptoms, personality traits, and emotional expression styles in a community sample of 151 participants aged 10–77 years, spanning four age groups: adolescents, young adults, middle-aged adults, and older adults. Methods: Psychological symptoms were assessed using the SCL-90, personality traits using the Big Five Inventory-2 (BFI-2), and emotional expression patterns were derived from facial expression recognition via a convolutional neural network (CNN) model. Kruskal–Wallis H tests were used to examine age-related differences. K-means cluster analysis was applied to identify emotional expression patterns, and logistic regression was used to construct a mental health risk screening model. Results: The young adult group (19–35 years) achieved the highest scores on the depression (M = 1.73) and anxiety (M = 1.61) dimensions, indicating a higher level of psychological distress during this life stage. Personality traits showed a significant developmental trajectory: neuroticism decreased with age (H(3) = 17.09, *p* < 0.001, η^2^ = 0.11), declining from 2.69 in the young adult group to 2.17 in the older adult group; conscientiousness increased with age (H(3) = 37.39, *p* < 0.001, η^2^ = 0.24), representing the most substantial age-related effect. K-means clustering identified three distinct emotional expression patterns: Cluster 1 was characterised by happiness, Cluster 2 by anger, disgust, and fear, and Cluster 3 by neutrality, sadness, and surprise. Cluster 2 exhibited the highest scores on neuroticism, anxiety, depression, and mood swings, and scored significantly higher than the other two clusters on interpersonal sensitivity, depression, anxiety, and hostility (*p* < 0.05). Mental health risk screening indicated that 26.5% of participants were classified as high-risk. Logistic regression analysis (AUC = 0.742) showed that neuroticism was the strongest predictor of elevated mental health risk (OR = 4.58), while extraversion (OR = 0.41) and conscientiousness (OR = 0.57) were significant protective factors. Conclusions: These findings provide exploratory evidence regarding age-related patterns of psychological symptoms and personality traits in a convenience sample and offer preliminary support for personality-based mental health risk screening. Notably, the SCL-90 was employed as a screening tool rather than for clinical diagnosis. Given the unequal age group sizes, particularly the small young adult subgroup, generalisability across the lifespan should not be assumed.

## 1. Introduction

Mental health and personality development issues continue to receive attention in contemporary psychology. The distribution of psychological symptoms across different life stages, age-related changes in personality dimensions, and inter-individual differences in emotional externalisation provide theoretical support for constructing mental health risk prediction models [1].

This study constructs an analytical framework based on three core theoretical perspectives. First, the Five-Factor Model (FFM) provides a stable operational foundation for measuring personality dimensions. Systematically developed by Costa and McCrae [2,3], this theory categorises personality traits into five dimensions: neuroticism, extraversion, openness, agreeableness, and conscientiousness. These dimensions demonstrate cross-cultural consistency and have been shown to predict important outcomes, including mental health status, social adaptability, and life satisfaction. For the present cross-age study, the Big Five Inventory-2 (BFI-2) was selected as the operationalization of the FFM. The BFI-2 is a 60-item revised version of the original BFI, developed by Soto and John [4], featuring improved item coverage, higher structural validity, and stronger discriminant validity across facets than its predecessor. Critically, the BFI-2 has demonstrated a stable factor structure and acceptable reliability across diverse age groups, from adolescents to older adults [5], making it well suited for lifespan comparative designs such as the present study.

Second, the theoretical foundation for emotional expression derives from Ekman’s Basic Emotions Theory [6,7,8]. Through cross-cultural research, Ekman established the existence of universal basic emotions (happiness, sadness, anger, fear, surprise, and disgust) that are expressed through specific facial expressions and are consistently recognised across cultures. This theory provides a theoretical basis for objectively measuring emotional externalisation patterns using facial expression videos, enabling the study to transcend traditional self-report methods and capture authentic individual differences in emotional expression. Accordingly, the present study targets facial emotional expression behaviour as its measurement construct, treating habitual expression patterns as behavioural phenotypes rather than as direct indicators of subjective felt emotion. This distinction is maintained throughout the manuscript.

The Lifespan Development Perspective serves as the foundation of this study [9,10,11]. Proposed by Baltes and colleagues, this theory emphasises that development is a lifelong process in which personality traits, emotion regulation abilities, and mental health exhibit dynamic trajectories across different ages. The perspective posits that development is multidimensional, multidirectional, and plastic; individuals face distinct developmental tasks at various life stages, and their personality characteristics and psychological functions continuously evolve through interactions among environmental, physiological, and social factors. This theoretical framework provides a rationale for this study’s design, spanning four age groups from adolescence to old age. It establishes a foundation for understanding age-related differences in the relationships among personality, emotion, and mental health.

From a lifespan development perspective, psychological symptom expression varies across age groups. Research has demonstrated that age of onset moderates subsequent symptom manifestations in major depressive disorder [12], while digital media use duration in adolescents correlates with mental symptom severity [13]. Normative studies using the SCL-90 scale have revealed temporal evolution in mental health status [14], with different age groups exhibiting distinct psychological problem profiles [15]. The SCL-90 has established Chinese normative data covering populations from early adolescence through late adulthood [16,17], and confirmatory factor analyses have supported the stability of its nine-factor structure in both clinical and community adolescent samples [18], as well as in large college-age samples [19]. Among older adults, the scale has demonstrated acceptable reliability. However, prior research suggests that the expression of psychological distress may differ somewhat from younger groups [20], a limitation acknowledged in the present study. Given the absence of a single cross-age measurement-invariance study spanning all four age groups, the SCL-90 was used as a uniform screening tool to enable cross-group comparisons of symptom severity rather than as a clinical diagnostic instrument, and group-level findings should be interpreted accordingly. Life stressors are considered important sources of psychological distress and somatization reactions [21], while individuals employing avoidance coping strategies often experience adverse health outcomes [22].

Personality dimensions change dynamically throughout the lifespan rather than remaining static [23]. The Big Five personality model, the most widely applied theoretical framework in personality psychology [24], provides an operational basis for exploring personality–health relationships—individuals with higher conscientiousness tend to exhibit more positive health behaviours. In contrast, those with elevated neuroticism often experience negative emotions and maladaptive behavioural tendencies. Recently, neural network technology has been applied to predict personality traits [25]. In older adults, personality dimensions mediate the relationship between childhood trauma and depressive symptoms [26] and are associated with cardiovascular disease risk [27].

Emotional externalisation characteristics constitute an important observational dimension in psychological symptom and personality research. Multiple studies have employed cluster analysis to explore heterogeneous structures within psychological symptoms, effectively identifying distinct depression subtypes [28]. Machine learning algorithms have demonstrated potential for distinguishing depression and anxiety symptom expression patterns [29], while similar computational techniques have quantified and analysed the effects of individual difference variables on treatment response [30].

Mental health risk assessment has emerged as a research focus in recent years. The existing literature indicates that social support levels correlate negatively with psychological problem severity [31], while psychological resilience serves a protective function in stressful situations [32]. Risk identification research has expanded from general populations to high-risk clinical groups [33], and machine learning technology has shown preliminary promise for psychosomatic disease classification and diagnostic support [34].

However, several gaps remain in the existing literature. First, most studies examine psychological symptoms, personality traits, or emotional expression in isolation, without simultaneously modelling their interrelationships across age groups [35]. Second, while cluster analysis has been applied to identify depression subtypes [28], its application to emotional expression patterns as a basis for mental health risk stratification remains underexplored. Third, existing risk prediction models based on personality traits have largely been developed in single age-group samples—predominantly young adults—limiting their generalizability across the lifespan [36]. Fourth, the SCL-90 has been used as a clinical diagnostic criterion in prior studies, whereas its application as a community screening tool across age-diverse samples warrants systematic examination. These gaps collectively point to the need for a multidimensional, cross-age analytical framework that integrates personality, emotional expression, and psychological symptom data for mental health risk screening.

Addressing these research gaps, this study simultaneously incorporates age, psychological symptoms, personality dimensions, and emotional externalisation characteristics into an analytical framework to construct a multidimensional mental health risk prediction model. Specifically, this study examines the dynamic evolution of psychological symptoms and personality dimensions across age stages, identifies potential risk subgroups based on emotional externalisation patterns, and establishes a risk prediction model to improve classification accuracy, thereby providing empirical evidence for early detection and intervention for mental health problems.

Building on the theoretical framework outlined above and the identified gaps in the literature, three directional hypotheses were formulated: (1) young adults would exhibit the highest levels of psychological symptoms, particularly depression, while conscientiousness would increase and neuroticism would decrease with age; (2) the subgroup characterised by negative emotional expressions (anger, disgust, and fear) would show significantly higher neuroticism and psychological symptom severity than subgroups characterised by positive or neutral expressions; (3) neuroticism would be the strongest positive predictor of mental health risk, while extraversion and conscientiousness would serve as significant negative predictors.

## 2. Materials and Methods

### 2.1. Participants and Procedure

This study recruited 198 participants through convenience sampling in the Hefei metropolitan area, Anhui Province, China, between May 2023 and May 2024. Participants were recruited via both online (social media announcements and online questionnaire platforms) and offline (community centres, retirement communities, and university campuses) channels. The study protocol was reviewed and approved by the Biomedical Ethics Committee of Hefei University of Technology (ethics number: HFUT20250110001H; approval date: 10 January 2025), and all participants provided written informed consent before participation. Given the convenience sampling approach, findings should be interpreted with caution regarding their generalizability to broader populations. Following data quality screening, 47 participants were excluded due to poor data quality (e.g., patterned or extreme response patterns, extensive missing values), yielding a valid sample of 151 participants (retention rate: 76.3%) aged 10–75 years with a relatively balanced gender composition.

Inclusion criteria were as follows: (1) age 10–77 years; (2) ability to independently understand and complete questionnaires; (3) no current mental illness diagnosis; and (4) voluntary participation with signed informed consent (obtained from guardians for minors). Exclusion criteria were as follows: (1) current psychiatric treatment or psychological counselling; (2) severe cognitive impairment affecting questionnaire comprehension; and (3) poor data quality (e.g., extensive missing values or patterned responses).

Participants represented diverse socioeconomic backgrounds with varying mental health levels. The assessment procedure comprised three components: a preliminary emotional state screening, a video recording of emotional behaviour externalisation, and questionnaire completion. Facial expressions and emotional externalisation characteristics were captured using high-definition video equipment, after which participants completed the Big Five Inventory-2 (BFI-2) and the Symptom Checklist-90 (SCL-90).

To examine lifespan developmental characteristics of psychological functioning, participants were stratified into four age groups following established conventions in lifespan developmental research and consistent with Erikson’s psychosocial developmental stages and Baltes’ lifespan framework [9,11], as well as Chinese normative SCL-90 studies [37]: adolescents (10–18 years, *n* = 36), young adults (19–35 years, *n* = 17), middle-aged adults (36–60 years, *n* = 59), and older adults (≥60 years, *n* = 39). Shapiro–Wilk normality tests indicated that multiple variables violated the normality assumptions; therefore, Kruskal–Wallis H tests were conducted to analyse age-group differences in psychological symptoms and Big Five personality dimensions (Table 1).

### 2.2. Measuring Tools

#### 2.2.1. Big Five Personality BFI-2 Scale

This study employed the Chinese version of the Big Five Inventory-2 (BFI-2), originally developed by Soto and John (2017) and subsequently translated and validated for Chinese populations by Zhang et al. (2022) [4,5]. The scale comprises 60 items rated on a 5-point Likert scale (1 = strongly disagree, 5 = strongly agree), measuring five personality dimensions: neuroticism, extraversion, openness, agreeableness, and conscientiousness. Each dimension comprises three facets, yielding 15 in total. Zhang et al. (2022) demonstrated strong psychometric properties of the Chinese BFI-2 across four Chinese samples [5], with Cronbach’s alphas ranging from 0.74 to 0.87 for the five dimensions. In the present study, Cronbach’s α coefficients were as follows: neuroticism (α = 0.715), extraversion (α = 0.600), openness (α = 0.619), agreeableness (α = 0.713), and conscientiousness (α = 0.815), all of which reached or approached acceptable reliability thresholds. The relatively lower coefficients for extraversion (α= 0.600) and openness (α = 0.619) are consistent with findings from prior Chinese BFI-2 validation studies, where these two dimensions also showed somewhat lower internal consistency [5]. This pattern likely reflects the broader, more heterogeneous nature of these constructs compared with neuroticism or conscientiousness. Effect size estimates for extraversion and openness should therefore be interpreted with caution, and the non-significant age-related findings for these dimensions may partly reflect measurement limitations, in addition to the small size of the young adult subgroup.

#### 2.2.2. SCL-90 Symptom Checklist

This study used the Symptom Checklist-90 (SCL-90), developed by Derogatis (2022) and translated into Chinese by Wang Zhengyu (2017) [16,38]. The scale comprises 90 items rated on a 5-point Likert scale (1 = none, 5 = severe) and measures nine symptom dimensions: somatization, obsessive–compulsive symptoms, interpersonal sensitivity, depression, anxiety, hostility, phobias, paranoid ideation, and psychoticism [29]. The Chinese version of the SCL-90 demonstrates good reliability and validity in Chinese populations, with a total Cronbach’s alpha of 0.97 and subscale coefficients ranging from 0.70 to 0.90 [17,39]. Jin Hua et al. (1986) established Chinese norms, providing a reference standard for mental health assessment in the general population [17].

It should be noted that this study employed the SCL-90 as a psychological symptom screening tool to identify individuals at risk of psychological distress rather than for clinical diagnosis. The study’s findings reflect associations among personality, emotion, and mental health in the general population and should not be equated with clinical diagnostic results. The sample in this study ranged in age from 10 to 77 years. The SCL-90 was applied uniformly for the following reasons: (1) this scale has been widely used across populations from adolescents aged 12 years and older to elderly individuals; (2) all participants aged 10–12 years (*n* = 10; 6 males, 4 females) completed the questionnaire under parental supervision to ensure accurate comprehension; and (3) the use of a standardised measurement tool ensured direct comparability of symptom severity across age groups, meeting the methodological requirements for cross-life-cycle comparative studies. To verify that including participants aged 10–11 years (*n* = 10) did not compromise data validity, all primary Kruskal–Wallis H analyses were repeated after their exclusion (*n* = 141). Results were highly consistent with the full-sample analyses across all 15 key variables, with virtually unchanged effect sizes (). The only marginal difference was on the phobia subscale (PHOB: *p* = 0.046 vs. 0.052), with identical effect sizes (=0.034 vs 0.033), confirming that their inclusion did not materially affect the conclusions (see Supplementary Table S1). Similarly, sensitivity analyses excluding these 10 participants confirmed robustness for Big Five age-group comparisons (neuroticism H = 19.582, *p* < 0.001; conscientiousness H = 37.615, *p* < 0.001, both unchanged in significance) and logistic regression discrimination (AUC = 0.724, 95% CI: 0.639–0.808, vs. full-sample AUC = 0.740). To further examine the psychometric comparability of the SCL-90 across age groups, inter-subscale Cronbach’s coefficients were computed separately for each of the four age groups using the nine core symptom subscales. Results indicated excellent and highly comparable internal consistency across all groups: adolescents (=0.942, 95% CI: 0.914–0.970), young adults (=0.948, 95% CI: 0.914–0.982), middle-aged adults (=0.933, 95% CI: 0.908–0.957), and older adults (= 0.918, 95% CI: 0.881–0.956). The narrow range of α values across groups (0.918–0.948) supports the comparability of SCL-90 measurement across the full age range of the present sample (see Supplementary Table S4).

#### 2.2.3. Video Data Collection and Data Output

All interviews followed a fixed question sequence lasting 35 to 50 min per participant, administered by trained counsellors using a standardised protocol, thereby ensuring consistent emotional elicitation conditions across participants. Facial expression videos were recorded using three high-definition cameras (1080-pixel resolution) positioned at different heights in a standardised, quiet interview environment. Participants were seated face-to-face with the interviewer at a distance of more than 1.5 m to ensure unobstructed frontal full-body capture. Recordings were captured at 25 Hz with an image resolution of up to 3264 × 2448 pixels and an automatic focus range of 5–50 mm. Fill-in light panels were employed when necessary to maintain consistent lighting conditions. Each video frame was subsequently preprocessed and cropped to 256 × 256 pixels before facial expression recognition.

Facial emotion recognition was conducted using a deep convolutional neural network (CNN) following the architecture described in Huang et al. (2023) [40]. Input frames were preprocessed and cropped to 256 × 256 pixels. A 2D convolution was applied to the three-channel RGB images, producing a 64-channel output feature map. Instance normalisation (InstanceNorm) and ReLU activation were subsequently applied, followed by multiple 3 × 3 2D convolutional layers with residual connections, yielding feature maps of size 128 × 128 × 256. Facial keypoint localisation was performed using two cascaded fourth-order Hourglass networks pre-trained on 68 facial landmarks, with multi-scale feature information fused via downsampling, upsampling, and residual modules. Feature fusion through 2D convolution, average pooling, max pooling, and dimensionality reduction yielded 1 × 4096 feature vectors, which were passed through a fully connected layer to output probability distributions (range: 0–1) for seven basic emotions: happiness, sadness, anger, fear, surprise, disgust, and neutrality. The model was trained on AffectNet, an open-source dataset comprising 420,000 sentiment-annotated images, achieving a per-class accuracy of 0.70 (AffectNet baseline: 0.58).

To improve recognition accuracy for Asian faces, transfer learning was applied using approximately 10,000 Asian facial images with semi-automatic annotation. The dataset was divided into a training set (70%) and a validation set (30%). A trained annotator first labelled all images according to established arousal and valence standards; samples with annotation confidence below 75% were then manually reviewed and relabelled to reduce label noise. The model’s overall per-class accuracy on AffectNet was 0.70, compared with the AffectNet baseline of 0.58, indicating meaningful improvement. Transfer learning further improved performance on Asian faces. The derived emotion scores should be treated as relative indicators of individual expression differences rather than ground-truth emotion classifications. Emotion probability scores were averaged across all frames per participant to derive individual-level emotion features. Emotional expressions were elicited naturally through the standardised semi-structured interview; participants were not instructed to perform or simulate specific emotions. Rather, the fixed question sequence covered topics relating to daily life, interpersonal experiences, and emotional well-being, designed to encourage authentic expression. Frame-level intraclass correlation coefficients (ICC = 0.287–0.589) confirmed that the aggregated scores captured meaningful between-person differences in habitual emotional expression tendencies. Emotion probability scores were derived from all video frames across the full interview session (35–50 min at 25 Hz, yielding approximately 52,500–75,000 frames per participant). Rather than counting discrete emotional episodes, the CNN model produced continuous probability scores for each of the seven emotion categories per frame, which were averaged across all frames to yield a single individual-level score per emotion dimension. This approach does not require participants to express a fixed number or type of emotions; individual differences in the resulting profiles were substantial and are precisely what the k-means clustering analysis was designed to capture. To mitigate potential demographic bias, no significant differences in emotion cluster membership were observed across age groups or gender (see Section 3.2.3), confirming that demographic factors did not confound clustering results.

### 2.3. Data Analysis

Data exclusion was based solely on response quality criteria (patterned responding and excessive missing values), not on distributional properties. Non-normality of the retained sample was addressed through the use of non-parametric tests throughout. This study used IBM SPSS 25.0 and R (version 4.4.2; R Core Team, 2024) for statistical analysis. Continuous variables were described as M ± SD, and categorical variables were expressed as percentages.

Emotion clustering was performed using the k-means method to analyse emotional expression data (0–1 probability values output by the CNN model) from 151 participants. Because all emotion variables were probability values output by the CNN model, with a uniform range of 0 to 1, they were naturally comparable in dimension and scale; therefore, no additional standardisation (e.g., z-score transformation) was performed. To verify the appropriateness of this decision, a k-means clustering analysis was conducted on z-score-standardised data. The results showed high consistency in cluster assignments before and after standardisation (Adjusted Rand Index = 0.76), indicating that the cluster structure was not affected by standardisation. K-means clustering is a distance-based algorithm that does not require multivariate normality assumptions. It does, however, rely on geometric assumptions: the algorithm minimises within-cluster squared Euclidean distances and implicitly favours spherical clusters of comparable size and variance, meaning that non-spherical or heterogeneous structures may not be well captured. The stability (mean ARI = 1.000) and silhouette width (mean = 0.299) of the present solution provide some empirical support for its adequacy, though alternative cluster structures cannot be ruled out. To assess the stability of the clustering solution, stability was assessed by comparing 100 runs, each using a distinct random seed and nstart = 1, against a reference solution (set.seed(42), nstart = 100) using the Adjusted Rand Index. The mean ARI was 1.000 (SD = 0.000), confirming perfect reproducibility of the three-cluster solution. The algorithm was optimised iteratively, alternating between the assignment and update steps until convergence. The optimal number of clusters was determined using the elbow and silhouette coefficient methods. For between-group comparisons, the Kruskal–Wallis H test was used to assess differences in psychological symptoms and personality traits across emotion clusters and age groups, as multiple variables violated the normality assumption as evaluated by the Shapiro–Wilk test. Post hoc comparisons were conducted using the Dunn test with Bonferroni correction, and effect sizes were reported as $\eta^{2}$. Bonferroni correction was consistently applied to all post hoc pairwise comparisons throughout the study, including those for personality dimensions and psychological symptom dimensions. Bonferroni-adjusted *p*-values are reported throughout. The mental health risk assessment was based on a comprehensive scoring system constructed from the SCL-90, integrating three criteria: a mean score ≥ 2.0 for any dimension, an overall mean score ≥ 2.0, and scores ≥ 1.5 for ≥ 3 dimensions. Each criterion was scored dichotomously (0/1), with comprehensive scores of 0–3 corresponding to low-, medium-, and high-risk levels. Differences in personality traits among risk groups were analysed using independent samples *t*-tests or Mann–Whitney U tests, with effect sizes reported as Cohen’s d. A binary logistic regression model was constructed with the Big Five personality traits as predictor variables (standardised) and mental health risk status as the dependent variable (0 = low-risk, 1 = medium-/high-risk). Unstandardized coefficients (B), standard errors (SE), and Wald $\chi^{2}$ values were reported. Model fit was assessed using Nagelkerke R^2^. ROC curve analysis was used to evaluate the model’s discriminatory ability, with AUC and 95% confidence intervals (CIs) reported. The optimal cutoff value was determined using the Youden index, and sensitivity, specificity, positive predictive value, and negative predictive value were calculated.

Participants were divided into four age groups: adolescents (10–18 years, *n* = 36), young adults (19–35 years, *n* = 17), middle-aged adults (36–60 years, *n* = 59), and older adults (≥ 60 years, *n* = 39). Gender did not significantly differ across the four age groups ($\chi^{2}$ = 2.074, df = 3, *p* = 0.557) or the three emotion clusters ($\chi^{2}$ = 1.288, df = 2, *p* = 0.525), and was therefore not included as a covariate in the main analyses. As a sensitivity analysis, Benjamini–Hochberg false discovery rate (FDR) correction was additionally applied across the 15 outcome variables. After correction, neuroticism (q = 0.005) and conscientiousness (q < 0.001) remained significant, confirming that the two primary age-related effects are robust to multiple comparison correction (see Supplementary Table S5 for full results). All tests were two-tailed with α = 0.05. To examine whether the unequal group sizes, particularly the small young adult subgroup (*n* = 17), affected the stability of the findings, a down-sampling sensitivity analysis was conducted. Participants from the three larger age groups were randomly sub-sampled to match the smallest group size (*n* = 17 per group), and Kruskal–Wallis H tests were repeated across 1000 random iterations. Conscientiousness remained significant in 100% of iterations and neuroticism in 81.7%, confirming the robustness of the two primary findings. Agreeableness, extraversion, and openness showed lower stability (14.5–40.0%) and should therefore be interpreted with caution (see Supplementary Table S6 for full results).

To verify that the clustering solution was not sensitive to variable scaling, k-means clustering was additionally applied to z-score standardised emotion probability scores. Cross-tabulation of individual cluster assignments revealed 92.1% agreement between the raw and standardised solutions (139 of 151 participants), and the dominant emotion profile of each cluster was preserved across both methods, confirming the robustness of the original three-cluster solution (see Supplementary Table S3 for cluster centroids under both methods).

To assess the degree to which frame-level emotion scores reflect stable between-person differences rather than momentary fluctuations, intraclass correlation coefficients (ICC; two-way mixed-effects model, absolute agreement, single measures) were computed for each of the seven emotion dimensions. ICC values quantify the proportion of frame-level variance attributable to stable individual differences, with higher values indicating greater consistency across frames within a recording session.

## 3. Results

### 3.1. Age-Related Differences in Psychological Symptoms and Personality Traits

#### 3.1.1. Age-Related Differences in Psychological Symptoms

The mean total SCL-90 scores for the four age groups were as follows: adolescents (10–18 years, *n* = 36, 18 males, 18 females), M = 1.39, SD = 0.36; young adults (19–35 years, *n* = 17, 10 males, 7 females), M = 1.57, SD = 0.44; middle-aged adults (36–60 years, *n* = 59, 25 males, 34 females), M = 1.32, SD = 0.29; older adults (≥ 60 years, *n* = 39, 21 males, 18 females), M = 1.32, SD = 0.27. All group mean scores fell below the clinical cutoff of 2.0, consistent with the expected distribution in a typical community sample and indicating that the SCL-90 effectively distinguishes between symptom levels in this cross-age sample.

The Kruskal–Wallis H test revealed significant age-related differences across multiple dimensions of psychological symptoms (Table 2). The young adult group (19–35 years) consistently exhibited the highest mean scores for depression (M = 1.73, SD = 0.56) and anxiety (M = 1.61, SD = 0.53), indicating higher levels of psychological distress at this developmental stage. In contrast, the older adult group exhibited relatively lower symptom levels across most dimensions, suggesting improved emotional well-being in later life.

Statistical analysis identified significant age effects for the following symptom dimensions: depression (DEP), H(3) = 8.44, *p* = 0.038, with young adults scoring significantly higher than other age groups; interpersonal sensitivity (I-S), H(3) = 8.21, *p* = 0.042, indicating that interpersonal sensitivity peaks in young adulthood; somatization (SOM), H(3) = 9.36, *p* = 0.025, with higher scores in both young and older adult groups; and phobic anxiety (PHOB), H(3) = 8.00, *p* = 0.046, showing age-related variation in anxiety manifestations.

#### 3.1.2. Age-Related Differences in Personality Traits

Highly significant age-related differences were observed in two key personality dimensions: neuroticism and conscientiousness (see Table 3). These findings are consistent with the maturation principle of personality development, which posits that individuals become more emotionally stable and socially responsible with age.

Neuroticism showed a significant decreasing trend throughout the lifespan (H(3) = 17.09, *p* < 0.001, $\eta^{2}$= 0.11, 95% BCa CI: 0.009–0.184). Adolescents (M = 2.56, SD = 0.59) and young adults (M = 2.69, SD = 0.44) exhibited higher neuroticism scores, while middle-aged (M = 2.45, SD = 0.49) and older adults (M = 2.17, SD = 0.44) showed significantly lower scores. This progressive decline in emotional instability reflects age-related enhancements in emotional regulation and psychological resilience. The effect size indicates that age accounts for approximately 11% of the variance in neuroticism scores, representing a medium effect.

Conscientiousness showed a progressive increase with age (H(3) = 37.39, *p* < 0.001, $\eta^{2}$=0.24, 95% BCa CI: 0.098–0.364), representing the most substantial age-related effect observed in this study. Adolescents exhibited the lowest conscientiousness scores (M = 3.44, SD = 0.58), followed by progressively higher scores in young adults (M = 3.45, SD = 0.59), middle-aged adults (M = 3.99, SD = 0.48), and older adults (M = 4.11, SD = 0.38). This developmental pattern reflects the increased self-discipline, organisation, and goal-oriented behaviour in adulthood. The effect size indicates that age accounts for approximately 24% of the variance in conscientiousness, suggesting a substantial developmental effect. It should be noted that these age-related patterns are based on cross-sectional comparisons within a single convenience sample and should be regarded as exploratory. Cross-sectional designs cannot establish developmental trajectories, and the unequal group sizes, particularly the young adult subgroup (*n* = 17), further limit the reliability of age-group comparisons involving that group. Other significant findings included agreeableness (H(3) = 9.85, *p* = 0.020, $\eta^{2}$ = 0.05, 95% BCa CI: 0.000–0.125), which showed a moderate increase with age, reflecting enhanced interpersonal warmth and cooperativeness in later life. The confidence interval for agreeableness includes zero, indicating that this effect should be interpreted with caution pending replication in larger samples. Extraversion and openness did not reach conventional significance (both H(3) = 7.65, *p* = 0.054, $\eta^{2}$= 0.05); however, these results should not be interpreted as evidence of the absence of an age-related change. An effect size of $\eta^{2}$ = 0.05 reflects a small-to-medium effect, and the directional patterns for both dimensions are consistent with normative developmental trajectories. Descriptively, extraversion showed a dip in the young adult group (M = 3.08) compared with adolescents (M = 3.35), middle-aged adults (M = 3.34), and older adults (M = 3.42). Openness showed a gradual declining trend from adolescence (M = 3.49) to older adulthood (M = 3.18). The failure to reach conventional significance most plausibly reflects limited statistical power given the small young adult subgroup (*n* = 17) rather than a true null effect, and replication with larger samples is needed.

### 3.2. Group Emotional Expression Clustering Phenomenon

#### 3.2.1. Emotional Clustering Analysis

**Figure 1:**
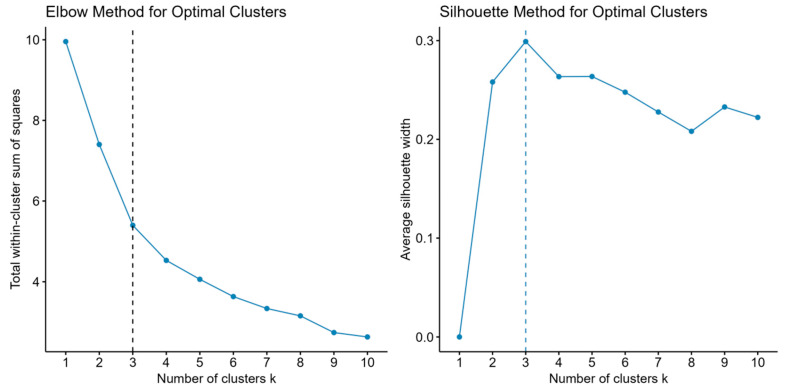
Optimal Number of Clusters Determined by the Elbow and Silhouette Methods.

Figure 1 visualises the methods used to determine the optimal number of clusters (K), employing two classic methods: the Elbow Method and the Silhouette Method [41]. Both methods indicate that K = 3 is the optimal number of clusters. To assess the robustness of the clustering solution, k-means clustering was repeated 100 times with different random initialisations. The mean Adjusted Rand Index (ARI) between the reference clustering and each of the 100 runs was 1.000 (SD = 0.000), indicating that the three-cluster solution was perfectly stable and reproducible across all initialisations.

Frame-level ICC analysis indicated moderate to fair between-person stability across the seven emotion dimensions (see Table 4). Anger showed the highest ICC (0.589), while fear showed the lowest (0.287). These values confirm that, after averaging across all frames, the derived emotion features capture a meaningful, stable component of individual expression style, supporting their use as person-level clustering variables.

**Figure 2:**
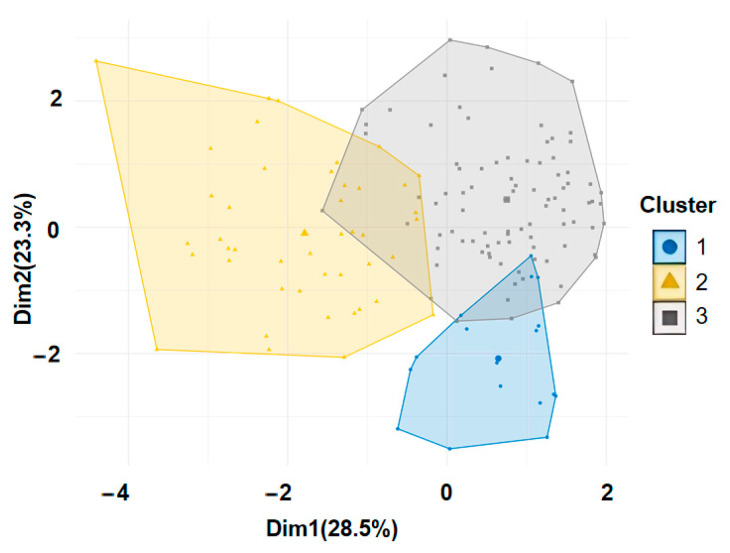
Visualisation of clustering results. Note: Average profile width = 0.299; Number of clusters = 3.

Figure 2 presents a visualisation of the clustering results, with an average silhouette width of 0.299, indicating reasonable partitioning. Per-cluster average silhouette widths were 0.430 (Cluster 1), 0.258 (Cluster 2), and 0.333 (Cluster 3). Cluster 2 showed the lowest cohesion, consistent with its larger size; only 2 of 44 members received negative silhouette values, indicating minimal boundary ambiguity. To facilitate presentation of the clustering structure in two-dimensional space, principal component analysis (PCA) was performed on the seven emotion variables (mean probabilities of happiness, sadness, anger, fear, surprise, disgust, and neutrality) used in k-means clustering. It should be noted that PCA was used solely for post hoc visualisation of the clustering results; the clustering itself was performed directly on the seven original emotion probability variables rather than on principal component scores. The 51.8% cumulative variance explained by PC1 and PC2 reflects the inherent dimensionality of the seven-emotion feature space rather than a limitation of the clustering analysis; the validity of the three-cluster solution is supported by independent metrics computed in the full seven-dimensional space, including perfect initialisation stability (mean ARI = 1.000, SD = 0.000) and a mean silhouette width of 0.299. In the two-dimensional plane formed by principal component 1 (28.5% variance explained) and principal component 2 (23.3% variance explained), the three clusters are marked with different colours, and their distributions are clearly visible. The 95% confidence ellipses reflect sample dispersion within each cluster. The boundaries between clusters are relatively distinct, demonstrating good discriminatory power, whereas the within-cluster distributions are relatively concentrated, indicating acceptable homogeneity.

#### 3.2.2. Characteristics of Emotional Expression in Cluster Grouping

**Figure 3:**
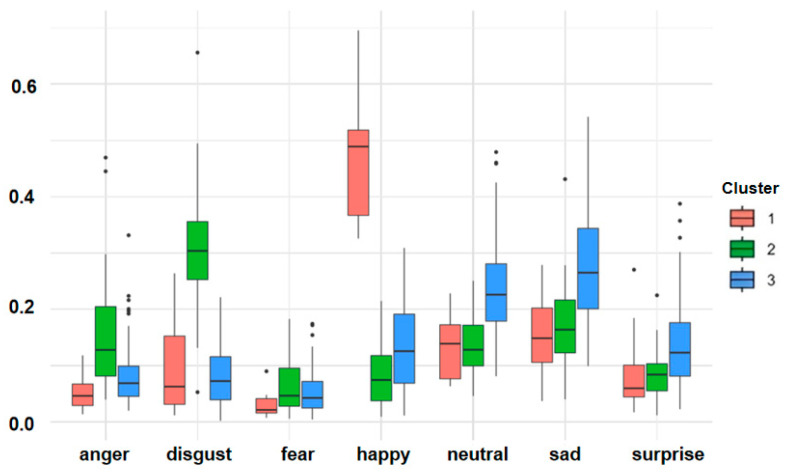
Emotion Clustering Results.

The three clusters comprised 17 (Cluster 1), 44 (Cluster 2), and 90 (Cluster 3) participants, respectively. Figure 3 shows the box plot of emotion distribution among clusters. Happiness was more prominent in Cluster 1, while anger, disgust, and fear were more prominent in Cluster 2, and neutrality, sadness, and surprise were more prominent in Cluster 3.

#### 3.2.3. Demographic Distribution of Cluster Groups

To eliminate potential confounding effects of demographic factors on emotion clustering results, gender and age distributions across the three clusters were compared. Chi-square analysis revealed no significant difference in gender distribution among the three clusters (χ^2^ = 1.288, df = 2, *p* = 0.525): Cluster 1 comprised 10 males and 7 females, Cluster 2 comprised 19 males and 25 females, and Cluster 3 comprised 45 males and 45 females. The Kruskal–Wallis H test similarly revealed no significant age differences among the three clusters (H(2) = 3.294, *p* = 0.193): mean ages were 52.9 ± 19.7 years for Cluster 1, 41.8 ± 20.7 years for Cluster 2, and 43.4 ± 21.6 years for Cluster 3. These results indicate that the three emotional expression patterns were not driven by gender or age differences.

### 3.3. Personality Trait Differences Across Emotional Clusters

#### 3.3.1. Fifteen Personality Dimensions

Across the 15 personality dimensions of the Big Five, significant statistical differences were observed in anxiety, depression, emotional volatility, social ability, self-confidence, energy, trust, orderliness, activity, and responsibility (*p* < 0.05 or adjusted *p* < 0.01). Post hoc tests revealed that Cluster 1 scored higher in social ability, self-confidence, trust, orderliness, and responsibility, while scoring lower in anxiety. Cluster 2 had higher scores in anxiety, depression, and emotional volatility, but lower scores in social ability, self-confidence, energy, trust, orderliness, activity, and responsibility. Cluster 3 showed higher scores in anxiety, energy, trust, orderliness, and responsibility, but lower scores in depression and emotional volatility.

#### 3.3.2. Five Personality Dimensions

Significant statistical differences in the Big Five personality traits were found between the emotional clusters in the neuroticism, agreeableness, and extraversion dimensions (*p* < 0.05). Post hoc tests showed that Cluster 2 had significantly higher neuroticism scores than Cluster 3 and Cluster 1 (*p* < 0.05). This indicates that individuals in Cluster 2, characterised by predominantly negative emotions, tend to have higher levels of emotional instability and vulnerability to stress.

In terms of agreeableness, Clusters 1 and 3 had significantly higher scores than Cluster 2 (*p* < 0.05), suggesting that individuals in these clusters are more cooperative, empathetic, and generally more socially adaptable. For extraversion, the order of scores was Cluster 1 > Cluster 3 > Cluster 2 (*p* < 0.05), with Cluster 1 scoring the highest. This implies that Cluster 1 individuals are more socially engaged and expressive. In contrast, Cluster 2 individuals, with lower extraversion, may exhibit more introverted tendencies and less outward emotional expression, as shown in Table 5.

#### 3.3.3. Differences in Personality Traits and Psychological Symptoms Across Emotional Clusters

We performed a nonparametric test with the emotional cluster as the independent variable to examine differences in psychological symptoms across the emotional clusters. The results showed that, except for obsessive–compulsive symptoms and somatisation, all other psychological symptoms exhibited significant differences across the different cluster groups. These included interpersonal sensitivity (*p* < 0.001), depression (*p* < 0.05), anxiety (*p* < 0.01), hostility (*p* < 0.05), phobia (*p* < 0.05), paranoid ideation (*p* < 0.05), psychosis (*p* < 0.001), and others (*p* < 0.001). Post hoc tests revealed that Cluster 2 showed higher psychological symptom scores compared to both Cluster 3 and Cluster 1. There were no significant inter-group differences between Cluster 3 and Cluster 1, except for the psychosis factor, where Cluster 3 scored higher than Cluster 1 on all other factors (Table 6).

### 3.4. Mental Health Risk Screening

#### 3.4.1. Screening Analysis of High-Risk Groups

This study developed a comprehensive risk assessment system based on the SCL-90 symptom spectrum to identify individuals at potential high risk of mental health problems, to provide targeted preventive interventions rather than for clinical diagnosis. The system uses a multi-criteria classification to determine risk levels. Its assessment algorithm integrates three complementary criteria from the SCL-90 symptom dimensions, as follows [37,40,43]: (1) average score ≥2.0 for any symptom dimension (moderate to severe symptoms); (2) overall average symptom score ≥2.0; (3) ≥3 symptom dimensions score ≥1.5 (mild to moderate symptoms). Each criterion is scored dichotomously (0 = not met, 1 = met). A comprehensive score of 0–3 corresponds to three risk levels: low, medium, and high (low-risk: 0 points; medium-risk: 1 point; high-risk: 2–3 points). The evaluation algorithm was applied to the full sample (*N* = 151), and the risk distribution results are as follows (see Table 7): low-risk: 84 people (55.6%); medium-risk: 27 people (17.9%); and high-risk: 40 people (26.5%).

To verify the differential effect of personality traits on risk categories, independent samples *t*-tests (or Mann–Whitney U tests) were performed on the Big Five personality dimensions for the low-risk group (*n* = 84) and the combined medium-to-high-risk group (*n* = 67). The results are shown in Table 8. Data showed that neuroticism had the most substantial differentiating effect (d = 0.81, significant effect), with significantly higher scores in the medium-to-high-risk group; extraversion (d = 0.56) and agreeableness (d = 0.54) showed moderate protective effects, conscientiousness showed a moderate protective effect (d = 0.44), and openness had no significant differentiating effect; all significant effects remained robust after Bonferroni multiple comparison correction (α = 0.01). These results clarify the personality traits associated with mental health risks, confirm neuroticism as a significant vulnerability factor, and highlight the importance of socioemotional competence for psychological resilience through the protective effects of extraversion and agreeableness.

#### 3.4.2. Predictive Modelling of Mental Health Risk

**Figure 4:**
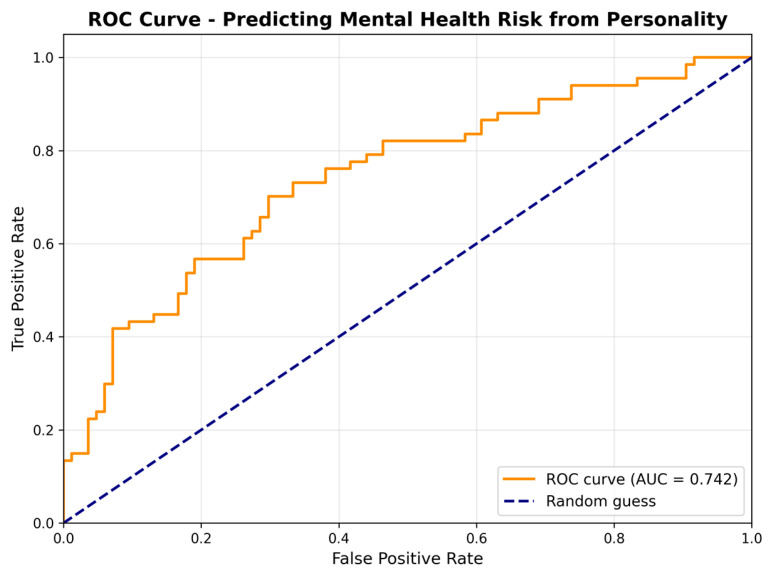
ROC curve of personality traits predicting mental health risk. Note: The model for predicting mental health risk based on personality traits has an AUC of 0.742, indicating moderate to good discriminative ability (the blue dashed line represents the random-guessing baseline).

**Figure 5:**
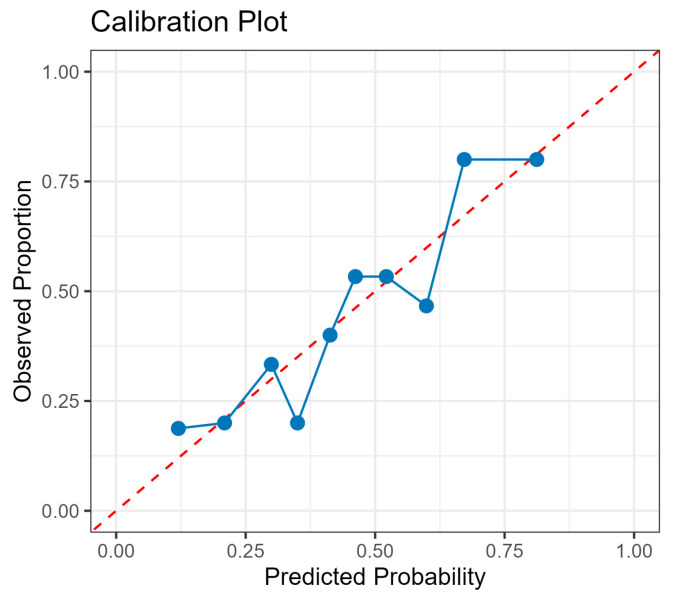
Calibration plot for the logistic regression model predicting mental health risk. Each point represents the mean predicted probability versus the observed proportion of medium/high-risk cases within a decile. The red dashed line indicates perfect calibration. Hosmer–Lemeshow χ^2^(8) = 4.811, *p* = 0.778.

This study constructs a logistic regression model to predict binary mental health risk status (0 = low-risk; 1 = medium-/high-risk) using the Big Five personality traits as simultaneous predictors, quantifying each trait’s independent contribution while controlling for cross-correlation and assessing overall classification accuracy. Receiver Operating Characteristic (ROC) curve analysis evaluates the model’s discriminative ability through threshold-independent performance assessment by plotting sensitivity against false positive rate (1—specificity) (Figure 4). The model demonstrates acceptable to good performance with an area under the curve (AUC) of 0.742 (95% CI: 0.663–0.821), indicating that it correctly ranks randomly selected high- and low-risk individuals in 74% of cases, significantly exceeding chance level (50%). At the optimal threshold of 0.444, the model achieves balanced performance metrics with 70.2% classification accuracy, 70.1% sensitivity, 70.2% specificity, 68.9% positive predictive value, and 71.4% negative predictive value, making it suitable for screening scenarios that require a balance between minimising false positives and false negatives. To assess internal validity, a 10-fold cross-validation was performed. The mean AUC across folds was 0.708 (95% CI: 0.642–0.774), closely approximating the full-sample AUC of 0.742, confirming the model’s stability and generalizability. Bootstrap internal validation (1000 iterations) yielded an optimism estimate of 0.032, producing an optimism-corrected AUC of 0.708, further corroborating the cross-validation estimate and indicating minimal overfitting. Variance inflation factor (VIF) values for all five predictors ranged from 1.26 to 1.91 (all < 2.0), indicating no multicollinearity concern. The Hosmer–Lemeshow test (χ^2^(8) = 4.811, *p* = 0.778) and a calibration plot (Figure 5) both indicate adequate fit between predicted probabilities and observed outcome rates (Table 9). Sensitivity analyses varying the three dichotomous SCL-90 risk criteria across loose, standard, and strict thresholds demonstrated that model discriminability remained acceptable to good across all specifications (see Table 10), supporting the robustness of the risk classification algorithm.

Standardised regression analysis clarified the independent predictive contributions of each personality dimension, controlling for the other traits (see Table 9). The results showed that neuroticism was the strongest predictor of increased risk (B = +1.523, *p* < 0.001), with the risk advantage ratio increasing 4.6-fold for every 1 standard deviation increase (OR = 4.58, 95% CI: 2.62–8.01); extraversion was a significant protective factor (B = −0.891, *p* = 0.003), with the risk advantage ratio decreasing by 59% for every 1 standard deviation increase (OR = 0.41, 95% CI: 0.23–0.73). Given the lower internal consistency of the extraversion subscale (α = 0.600), the magnitude of this estimate may be attenuated by measurement error and should be interpreted accordingly. Conscientiousness had a moderate protective effect (B = −0.562, *p* = 0.021), with the risk advantage ratio decreasing by 43% for every 1 standard deviation increase (OR = 0.57, 95% CI: 0.35–0.93); the pleasantness tendency protective effect did not reach the traditional significance level (B = 0.398, *p* = 0.136), and the independent contribution of openness was the smallest (B = 0.152, *p* = 0.493). The non-significant finding for openness is consistent with its lower internal consistency (α = 0.619), which may have attenuated the detection of any true effect. The overall model test was significant (χ^2^(5) = 47.82, *p* < 0.001), with Nagelkerke R^2^ = 0.368.

This study delves into the complex relationships among personality traits, emotional expression, and mental health, with a particular focus on the moderating role of age in these processes and on how these findings can be applied to construct an effective mental health risk screening model. The results not only reveal the dynamic changes in personality traits with age and their profound impact on mental health, but also identify the association between different emotional expression patterns and the risk of psychological symptoms through emotion clustering analysis, providing a new perspective for the early identification and intervention of mental health issues.

## 4. Discussion

The relationship between personality dimensions, emotional externalisation, and mental health constitutes the core focus of this study, with particular attention to the moderating role of age. Neuroticism scores exhibited a statistically significant decreasing trend across age groups (η^2^ = 0.11), with higher scores in adolescent and young adult groups and significantly lower scores in middle-aged and elderly groups. This trajectory suggests that emotional stability and psychological resilience may increase with age [44]. The age effect on conscientiousness was the most prominent (η^2^ = 0.24). Adolescent participants scored lowest on this dimension, with scores gradually increasing across age groups and reaching peak levels in the elderly group. This pattern aligns with the continuous reinforcement of self-discipline, organisation, and goal-directed behaviour throughout adulthood [45].

Notably, young adults aged 19–35 scored highest across all groups on depression, anxiety, and interpersonal sensitivity dimensions. Existing literature identifies this developmental period as a high-incidence stage for psychological distress [46]. Socioemotional selectivity theory provides a possible explanatory framework for improved emotional well-being in older adults, suggesting that individuals increasingly optimise their emotional regulation and expression as they age, actively focusing on positive emotions while avoiding negative stimuli [47].

The sample spanned ages 10–77, with symptom scores across age groups showing characteristics consistent with developmental stages. The young adult group (19–35 years) exhibited the highest depression (M = 1.73) and anxiety (M = 1.61) scores, consistent with stress associated with developmental tasks, including academic achievement, career establishment, and intimate relationship formation. The elderly group’s somatisation score (M = 1.37) paralleled that of the young adult group, potentially reflecting ageing-related chronic physical symptoms. The adolescent group showed relatively elevated interpersonal sensitivity (M = 1.62) and hostility (M = 1.42), consistent with peer relationship sensitivity characteristic of this developmental period [48]. Total mean scores across groups exhibited similar ranges (1.32–1.57), indicating stable SCL-90 measurement performance across this age-diverse sample. It should be noted that the young adult group comprised only 17 participants; all findings specific to this group, including the elevated depression and anxiety scores, should be treated as directional rather than definitive and require replication in larger young adult samples before broader conclusions can be drawn. Future research should further examine age as a moderating variable or conduct controlled verification using age-specific assessment tools for adolescents aged 10–18. Extraversion and openness showed marginal but non-significant effects (H(3) = 7.65, *p* = 0.054, η^2^ = 0.05). Given that η^2^ = 0.05 corresponds to a small-to-medium effect, these results deserve brief substantive comment. The lower extraversion score in the young adult group (M = 3.08) relative to the remaining three groups may reflect role-related pressures specific to early adulthood, with scores recovering thereafter. The gradual decline in openness from adolescence to older adulthood is directionally consistent with normative personality development research [1]. Both non-significant results are likely attributable to the small young adult subgroup (*n* = 17) rather than a true absence of age-related change, and replication with larger samples is needed.

Cluster analysis revealed three distinct emotional externalisation patterns, each with statistically significant associations with the Big Five personality dimensions. It should be emphasised that the CNN-derived probability scores represent observable facial expression behaviour rather than direct measurements of internal emotional experience. The cluster labels describe habitual expression patterns as behavioural phenotypes and should not be interpreted as indicators of subjectively felt emotion. Accordingly, the associations reported below are between facial expression patterns and personality or symptom profiles, not between felt emotion and psychopathology. Importantly, no significant differences emerged among clusters regarding gender (*p* = 0.525) or age (*p* = 0.193), suggesting that the identified emotional externalisation patterns are not attributable to demographic confounds, though the cross-sectional design does not permit inference about the temporal stability or trait-like nature of these patterns.

Cluster 1, characterised predominantly by positive emotions, showed higher scores for extraversion, agreeableness, and conscientiousness, along with relatively lower neuroticism. Cluster 2, dominated by negative emotions, showed significantly elevated neuroticism, reduced extraversion and agreeableness, and heightened anxiety, depression, and mood instability in the present sample. Cluster 3, characterised primarily by neutral, sad, and surprised expressions, occupied an intermediate position but outperformed Cluster 2 on energy and motivation indices. It should be noted that the extraversion subscale showed lower internal consistency in the present sample (α = 0.600); the extraversion differences observed across clusters may therefore partly reflect measurement imprecision and should be interpreted with caution.

The present data indicated an association between emotional externalisation patterns and mental health status. Cluster 2 participants scored higher than other groups across multiple SCL-90 dimensions, including interpersonal sensitivity, depression, anxiety, hostility, paranoid ideation, and psychoticism, suggesting that habitual negative emotional expression was associated with elevated risk for multiple psychological symptoms [49]. It is worth emphasising that Cluster 2 is characterised not by sadness but by a co-occurring pattern of anger, disgust, and fear. The association between this specific combination and elevated depression and anxiety scores is not self-evidently circular, as anger and disgust are not definitional components of depression in standard diagnostic criteria. The contribution of the present clustering analysis lies in identifying this multivariate behavioural phenotype and documenting its co-occurrence with elevated neuroticism, reduced extraversion and agreeableness, and higher scores across multiple SCL-90 dimensions within this sample. Conversely, Cluster 1 participants exhibited lower overall psychological symptom levels, consistent with existing research demonstrating an association between positive emotions and lower mental health symptom levels [50]. These cluster-based association, however, were derived from a single convenience sample from one geographic region and have not been validated in independent samples. The generalizability of this three-cluster typology to other populations or cultural contexts cannot be assumed from the present data alone.

The mental health risk screening system constructed in this study stratified participants into three risk levels: low-risk (55.6%), medium-risk (17.9%), and high-risk (26.5%). In distinguishing low-risk from medium- and high-risk groups, personality dimensions showed notable discriminative power within this sample. Neuroticism exhibited the most prominent effect (d = 0.81, large effect size), while extraversion (d = 0.56) and agreeableness (d = 0.54) both showed moderate protective effects, with conscientiousness demonstrating a moderate protective impact (d = 0.44). The logistic regression model achieved acceptable-to-good classification performance (AUC = 0.742, 95% CI: 0.663–0.821), correctly ranking randomly selected pairs of high- and low-risk individuals in 74% of cases. Regarding predictor weights, neuroticism was the strongest predictor of risk in this sample (OR = 4.58), while extraversion (OR = 0.41) and conscientiousness (OR = 0.57) were associated with lower risk. These findings provide a preliminary empirical basis for early identification; however, the AUC of 0.742 is an internal estimate, no external validation dataset was available, and the 10-fold cross-validation mean AUC (0.708) should be regarded as the more conservative performance indicator. The model’s discriminative ability in independent samples from other regions, as well as the predictive weights of individual personality dimensions across different socioeconomic and cultural contexts, remains to be established.

These clustering results hold potential application value across multiple mental health service domains. However, the following implications are preliminary and contingent on independent validation of the CNN-derived emotion features in future studies. First, regarding screening and stratification, the three emotional externalisation patterns can serve as preliminary screening indicators of mental health risk within routine psychological assessment protocols. Specifically, individuals classified into Cluster 2 (dominated by anger, disgust, and fear) showed elevated symptom burden in the present sample, suggesting this pattern may warrant closer attention in screening contexts. Whether this association is sufficiently stable and generalisable to support routine clinical flagging, however, requires prospective validation in independent samples before any applied use. Compared with traditional self-report scales, facial expression video-based emotion recognition offers advantages, including non-invasiveness and reduced social desirability bias, proving particularly suitable for populations with limited self-report capabilities (e.g., adolescents or cognitively impaired elderly individuals).

Second, regarding differentiated intervention design, cluster results inform personalised intervention planning. For Cluster 2 individuals, their personality profile—high neuroticism, low extraversion, and low agreeableness—suggests that interventions centred on emotion regulation skills training may be worth prioritising. However, this hypothesis requires prospective testing, including cognitive reappraisal strategies and mindfulness-based stress reduction [51]. For Cluster 3 individuals (dominated by neutral and sad emotions), whose symptom levels fall between Clusters 1 and 2, preventive interventions emphasising social skills training and positive emotion activation may be associated with lower risk progression, pending longitudinal validation. For Cluster 1 individuals (predominantly positive emotions) who exhibit favourable mental health, the focus should be on maintaining and enhancing psychological resilience by strengthening existing protective factors.

Third, concerning longitudinal monitoring, emotion clustering can serve as a baseline marker for dynamic tracking. Migration of an individual’s emotional externalisation pattern from Cluster 1 or 3 to Cluster 2 across time points may co-occur with deteriorating mental health; whether such migration temporally precedes symptom worsening remains to be established in longitudinal studies. This dynamic monitoring approach, based on changes in emotional externalisation patterns, aligns with contemporary mental health services’ increasing emphasis on prevention and personalisation.

Finally, this study’s integrated analysis of emotion clustering with personality dimensions and SCL-90 symptom data demonstrates that emotional externalisation patterns represent not isolated behavioural manifestations but rather systematic correlates of personality structure and psychological symptom profiles. This finding provides a preliminary empirical foundation for constructing a three-dimensional “emotional expression–personality traits–psychological symptoms” joint assessment framework. Future research should explore the feasibility of embedding emotion-clustering labels into multimodal mental health risk prediction models to enhance classification accuracy.

The most consequential limitation concerns external validity. First, all participants were recruited through convenience sampling within the Hefei metropolitan area; findings should not be generalised to broader Chinese or international populations without independent replication. Second, the young adult subgroup (*n* = 17) was substantially smaller than the other three groups; all results specific to this age group should be regarded as preliminary. Third, the three-cluster emotional expression typology was both derived and evaluated within the same sample and has not been externally validated; its stability across different populations remains unknown. Fourth, the logistic regression risk model lacks external validation, and the AUC of 0.742 reflects internal fit only, which is likely to overestimate true discriminative performance in new samples. Fifth, the CNN-based emotion inference pipeline was not validated on a held-out subset of the present sample; therefore, the absolute accuracy of the derived emotion scores in this community population remains unknown. Per-participant averaging across 52,500–75,000 frames and frame-level ICC values of 0.287–0.589 provide partial assurance that the aggregated scores reflect stable individual differences, and the cluster-based conclusions rest on relative distributional patterns rather than the accuracy of any individual classification. Independent external validation remains a priority for future work. This study exhibits several design and implementation limitations. The cross-sectional design precludes causal inference about the relationships among personality dimensions, emotional externalisation, and psychological symptoms. In particular, whether negative expression patterns precede symptom onset or are consequences of existing distress cannot be determined from the present data, and this constitutes a priority question for future longitudinal research. Elucidating the temporal dynamics and interaction mechanisms of these variables requires longitudinal designs [51]. Additionally, all measures were collected at a single time point, whereas both psychological symptoms and emotional expression are known to vary over time and across situational contexts. The extent to which the identified patterns reflect stable individual characteristics rather than state-dependent responses warrants careful consideration. Frame-level ICC analysis provided partial empirical support: ICC values ranged from 0.287 (fear) to 0.589 (anger), indicating that a moderate proportion of frame-level variance is attributable to stable between-person differences [42]. Averaging probability scores across all frames of a 35–50 min interview further attenuates momentary noise, aligning with the operationalisation of affective traits as temporally aggregated expression tendencies [52]. Nevertheless, the relatively lower ICC for fear (0.287) suggests that fear-related cluster differences should be interpreted with particular caution, and longitudinal assessment across multiple occasions would be needed to establish the test–retest stability of the identified expression patterns directly.

The sample size of 151 participants and the severely unequal age group distribution (young adult subgroup *n* = 17) constitute a significant limitation. No a priori power analysis was conducted before data collection. Post hoc sensitivity analysis indicated that with *N* = 151, four groups, α = 0.05, and power = 0.80, the study was adequately powered to detect effects of η^2^ ≥ 0.069 for age-group comparisons; both primary findings (neuroticism η^2^ = 0.11; conscientiousness η^2^ = 0.24) exceeded this threshold. For the logistic regression model, the minimum detectable effect was f^2^ = 0.088, well below the observed f^2^ = 0.582 (Nagelkerke R^2^ = 0.368). However, statistical power was insufficient to detect small effects reliably, and findings for dimensions with smaller effect sizes should be interpreted with caution. Future studies should conduct a priori power analyses and recruit 30–40 participants per age group. Although the down-sampling sensitivity analysis confirmed the robustness of the two primary findings, the small subgroup size reduces power for dimensions with smaller effect sizes. The lower internal consistency of the extraversion and openness subscales (=0.600 and 0.619, respectively) may have attenuated effect size estimates for these two dimensions. Findings involving extraversion and openness should therefore be interpreted with caution, and future studies should consider alternative scoring approaches or report facet-level reliability to better characterise the psychometric properties of these dimensions in Chinese samples. Socioeconomic and educational data were not systematically collected in the present study; occupational categories served as a partial proxy for SES and are reported in Table 1. The absence of formal education-level data is acknowledged as a limitation, as educational attainment may independently influence both personality trait expression and psychological symptom severity. Future studies should incorporate standardised measures of education and SES to enable covariate adjustment and more rigorous assessment of socioeconomic confounding. Incorporating multicenter data would further enhance generalizability [53]. Regarding the cluster analysis specifically, while the three-cluster solution demonstrated perfect reproducibility across 100 random initialisations (ARI = 1.000), the generalizability of the identified emotional expression patterns to broader populations warrants caution, and replication in larger multicenter samples is recommended.

The study employed only the SCL-90 self-report scale for symptom screening, lacking validation from gold standard diagnostic tools such as structured clinical interviews (e.g., SCID), precluding distinction between clinically diagnosed cases and subclinical symptom presentations. Self-report scales remain susceptible to social desirability bias and self-awareness limitations, potentially overestimating or underestimating true mental health status. Additionally, SCL-90 emphasises symptom severity assessment without adequately examining functional impairment—a critical clinical diagnostic criterion. More specifically, the following subsequent steps are suggested. First, a pre-registered longitudinal study following the same participants for at least two time points could directly assess whether emotional expression cluster membership predicts subsequent changes in psychological symptoms. Second, future studies should use structured clinical interviews such as the Structured Clinical Interview for DSM Disorders, SCID, in conjunction with SCL-90, to establish the convergent validity between the risk classification system and the gold standard diagnostic criteria. Third, multimodal physiological measures, including heart rate variability, skin conductance, and EEG, should be integrated to provide objective, non-self-reported indicators of emotional state and complement facial expression data [54]. The application of adult-normed instruments (SCL-90 and BFI-2) to participants aged 10–12 years (*n* = 10) represents an additional methodological concern. Constructs such as psychoticism and aesthetic sensitivity have limited developmental validity at this age, and reliance on parental supervision for item comprehension may have introduced response bias. Although the sensitivity analyses described in Section 2.2 confirmed that excluding these participants did not materially alter the primary findings (Big Five age-group comparisons: neuroticism H = 19.582, *p* < 0.001; conscientiousness H = 37.615, *p* < 0.001; logistic regression AUC = 0.724, 95% CI: 0.639–0.808), future studies should employ age-appropriate instruments for participants below age 13.

The emotional externalisation assessment relied primarily on facial expression analysis. Furthermore, although standardised recording conditions were maintained, the structured interview setting may not fully capture spontaneous emotional expressions in naturalistic contexts, potentially limiting the ecological validity of the emotion recognition results. Three factors limit the external validity of the present findings. First, the sample was recruited from a single province in eastern China, and cultural norms surrounding emotional expression and help-seeking behaviour may differ substantially across regions and countries, warranting caution in generalising findings to non-Chinese populations. Second, the age distribution was unequal across groups, with the young adult subgroup substantially underrepresented (*n* = 17); findings for this age group should therefore be interpreted with particular caution. Third, the CNN emotion recognition model was pre-trained on AffectNet and fine-tuned on Chinese facial images; its recognition accuracy for other ethnic groups has not been validated, and cross-ethnic application of the emotion clustering results requires independent replication. A conceptual limitation of the present study is that facial expression recognition captures expressed emotion rather than felt emotion. Facial expressions can be voluntarily masked or faked, and the two need not correspond; a point well illustrated in clinical contexts where individuals may maintain positive facial affect despite underlying distress. The present study, therefore, characterises habitual facial expression patterns as behavioural phenotypes in their own right rather than as proxies for internal emotional states. Future research incorporating self-reported momentary affect alongside facial expression data would allow direct examination of the expression–experience correspondence within this population. A further limitation concerns the scope and nature of emotions assessed. Basic emotions represent prototypical high-intensity affective states and may not adequately capture the full range of everyday emotional experience. More specifically, the seven basic emotions captured by the CNN model do not include self-conscious emotions such as guilt, shame, and helplessness, which are theoretically more proximal to the aetiology of depression and cannot currently be captured by automated facial recognition systems. The associations observed between Cluster 2 and psychological symptoms should therefore be interpreted as concurrent correlates rather than etiological indicators. Future studies should consider complementing categorical emotion recognition with dimensional approaches (e.g., valence–arousal circumplex models) to provide a more comprehensive characterisation of affective expression in naturalistic settings. The use of frame-level mean probability scores as clustering inputs represents a further methodological limitation. Although ICC analysis indicated moderate frame-level stability for the primary clustering emotions (anger ICC = 0.589, disgust ICC = 0.458, neutral ICC = 0.448, sad ICC = 0.431), the arithmetic mean cannot distinguish brief, intense emotional episodes from sustained, mild ones. Future studies should extract the standard deviation and the proportion of time spent in the dominant emotion as complementary clustering features to better capture emotional variability and state duration.

Evaluating the effectiveness of mental health interventions constitutes another critical future research direction. The mechanisms by which interventions such as emotion regulation training and cognitive-behavioural therapy influence emotional externalisation patterns and mental health warrant further investigation [55]. This research would provide an empirical foundation for transitioning mental health services from reactive to preventive models and advancing the development of personalised interventions.

## 5. Conclusions

This study provides exploratory evidence that personality traits and facial emotional expression patterns are associated with psychological symptom profiles across age groups in a community sample. However, the cross-sectional design and unequal group sizes preclude conclusions about developmental trajectories or lifespan generalisability. Individuals whose emotional expression was dominated by negative affect (anger, disgust, and fear) exhibited elevated neuroticism and greater psychological symptom burden, while neuroticism, extraversion, and conscientiousness together provided acceptable screening discrimination for mental health risk (AUC = 0.742). These findings offer preliminary empirical support for personality-informed mental health screening and suggest that integrating facial emotion recognition with Big Five assessment may contribute to early identification of at-risk individuals. However, this possibility remains to be tested in prospective designs with independent samples. All findings should be interpreted within the constraints of the cross-sectional design, which precludes causal inference, and the convenience sample drawn from a single region, which limits generalisability. Longitudinal replication with structured clinical interview validation is needed before these results can inform clinical practice.

## Figures and Tables

**Table 1:** Demographic Characteristics of Each Age Group.

Age Group	Sample Size (n)	Mean Age (SD)	Gender Distribution	Age Range	Occupation
Adolescents (10–18 years)	36	15.03 ± 2.60	18 males/18 females	10–18	Students (100%)
Young Adults (19–35 years)	17	27.35 ± 4.40	7 males/10 females	19–35	Students, employees, self-employed
Middle-aged (36–60 years)	59	48.93 ± 7.05	34 males/25 females	36–60	Workers, employees, self-employed
Elderly (60 years and older)	39	70.49 ± 4.01	18 males/21 females	60–81	Retired (100%)

Note: Gender distribution was balanced across the four age groups ($\chi^{2}$ = 2.074, df = 3, *p* = 0.557) and across the three emotional expression clusters ($\chi^{2}$ = 1.288, df = 2, *p* = 0.525), indicating that gender did not introduce confounding effects in either analysis.

**Table 2:** Age-Related Differences in Psychological Symptom Dimensions.

Symptom Dimension	Adolescent Group	Young Adult Group	Middle-Aged Group	Older Group	H-Value	*p*-Value
Somatization	1.19 ± 0.29	1.37 ± 0.30	1.27 ± 0.30	1.37 ± 0.34	9.36	0.025 *
Obsessive Compulsive Symptoms	1.49 ± 0.42	1.69 ± 0.47	1.42 ± 0.39	1.51 ± 0.38	4.89	0.180
Interpersonal Sensitivity	1.62 ± 0.53	1.81 ± 0.64	1.42 ± 0.43	1.40 ± 0.35	8.21	0.042 *
Depression	1.41 ± 0.46	1.73 ± 0.56	1.38 ± 0.36	1.48 ± 0.39	8.44	0.038 *
Anxiety	1.31 ± 0.40	1.61 ± 0.53	1.30 ± 0.33	1.28 ± 0.30	5.11	0.164
Hostility	1.42 ± 0.46	1.52 ± 0.59	1.35 ± 0.40	1.20 ± 0.27	6.26	0.100
Phobic Anxiety	1.39 ± 0.48	1.43 ± 0.52	1.16 ± 0.27	1.21 ± 0.39	8.00	0.046 *
Paranoid Ideation	1.41 ± 0.46	1.50 ± 0.58	1.32 ± 0.37	1.25 ± 0.34	2.82	0.420
Psychoticism	1.29 ± 0.37	1.44 ± 0.39	1.24 ± 0.28	1.23 ± 0.31	5.51	0.138

Note: Values are presented as mean ± SD. * *p* < 0.05.

**Table 3:** Age-Related Differences in the Big Five Personality Traits.

Personality Trait	Adolescent Group	Young Adult Group	Middle-Aged Group	Older Group	H-Value	*p*-Value	η^2^	95% BCa CI
Neuroticism	2.56 ± 0.59	2.69 ± 0.44	2.45 ± 0.49	2.17 ± 0.44	17.09	<0.001 **	0.11	0.009–0.184
Agreeableness	3.86 ± 0.48	3.88 ± 0.33	4.04 ± 0.38	4.16 ± 0.40	9.85	0.020 *	0.05	0.000–0.125
Conscientiousness	3.44 ± 0.58	3.45 ± 0.59	3.99 ± 0.48	4.11 ± 0.38	37.39	<0.001 **	0.24	0.098–0.364
Openness	3.49 ± 0.59	3.31 ± 0.42	3.36 ± 0.46	3.18 ± 0.41	7.65	0.054	0.03	0.000–0.090
Extraversion	3.35 ± 0.51	3.08 ± 0.36	3.34 ± 0.44	3.42 ± 0.39	7.65	0.054	0.03	0.000–0.087

Note: Values are presented as mean ± SD. η^2^ was computed using the formula (H − k + 1)/(N − k). 95% CIs were estimated via bias-corrected and accelerated (BCa) bootstrap with 2000 iterations. * *p* < 0.05; ** *p* < 0.01.

**Table 4:** Intraclass Correlation Coefficients (ICC) for Frame-Level Emotion Probability Scores Across Seven Dimensions.

Emotion	ICC	95% CI Lower	95% CI Upper	Reliability Level
Anger	0.589	0.532	0.649	Moderate
Disgust	0.458	0.400	0.523	Moderate
Neutral	0.448	0.390	0.513	Moderate
Sad	0.431	0.373	0.496	Moderate
Surprise	0.365	0.310	0.429	Fair
Happy	0.346	0.292	0.409	Fair
Fear	0.287	0.237	0.347	Fair

Note: Reliability levels follow Koo & Li (2016) [42]: Fair = ICC < 0.50; Moderate = 0.50 ≤ ICC < 0.75.

**Table 5:** Non-Parametric Tests and Post Hoc Tests of Personality Traits.

	Non-Parametric Test		Post Hoc Test Comparisons						
	*X^2^*	*p*	①–②		①–③		②–③		
			d [95% CI]	*p*	d [95% CI]	*p*	d [95% CI]	*p*	
15 Personality Dimensions									
Anxiety	13.20	0.001 **	−0.37 [−1.11, 0.32]	<0.001 ***	−0.23 [−0.88, 0.35]	0.003 **	0.10 [−0.21, 0.43]	0.413	②, ③ > ①
Depression	25.04	<0.001 ***	−0.60 [−1.19, −0.07]	<0.001 ***	−0.28 [−0.78, 0.21]	0.195	0.32 [−0.05, 0.69]	<0.001 ***	② > ①, ③
Emotional Volatility	11.73	0.003 **	−0.09 [−0.56, 0.34]	0.006 **	0.02 [−0.32, 0.37]	0.401	0.10 [−0.25, 0.46]	0.006 **	② > ①, ③
Social Ability	55.78	<0.001 ***	0.28 [−0.31, 0.88]	<0.001 ***	0.17 [−0.39, 0.72]	0.004 **	−0.12 [−0.49, 0.25]	<0.001 ***	① > ③ > ②
Self-Confidence	29.30	<0.001 ***	0.15 [−0.34, 0.68]	<0.001 ***	0.07 [−0.38, 0.48]	0.002 **	−0.08 [−0.44, 0.29]	<0.001 ***	① > ③ > ②
Energy	9.33	0.009 **	0.11 [−0.50, 0.73]	0.055	−0.05 [−0.57, 0.46]	1.000	−0.16 [−0.49, 0.18]	0.005 **	③ > ②
Curiosity	3.19	0.203	−0.10 [−0.71, 0.45]		0.11 [−0.38, 0.62]		0.21 [−0.13, 0.58]		
Aesthetic Sensitivity	1.40	0.496	−0.34 [−0.96, 0.28]		−0.27 [−0.85, 0.30]		0.06 [−0.30, 0.41]		
Creativity	0.90	0.638	−0.16 [−0.79, 0.41]		0.07 [−0.43, 0.54]		0.23 [−0.12, 0.58]		
Empathy	4.75	0.093	0.56 [0.01, 1.19]		0.28 [−0.24, 0.82]		−0.30 [−0.68, 0.09]		
Respect	3.77	0.152	0.27 [−0.27, 0.80]		−0.11 [−0.58, 0.36]		−0.37 [−0.73, −0.01]		
Trust	13.36	0.001 **	0.75 [0.24, 1.32]	0.005 **	0.23 [−0.19, 0.67]	0.521	−0.43 [−0.76, −0.10]	0.002 **	①, ③ > ②
Orderliness	10.61	0.005 **	0.28 [−0.29, 0.91]	0.009 **	0.19 [−0.27, 0.66]	0.463	−0.06 [−0.41, 0.27]	0.008 **	①, ③ > ②
Activity	6.47	0.039 *	0.35 [−0.23, 0.98]	0.077	0.18 [−0.29, 0.67]	0.931	−0.13 [−0.48, 0.20]	0.031 *	③ > ②
Responsibility	8.91	0.012 *	0.47 [−0.13, 1.12]	0.022 *	0.29 [−0.21, 0.82]	0.623	−0.14 [−0.49, 0.21]	0.014 *	①, ③ > ②
Big Five Personality Traits									
Neuroticism	16.88	<0.001 ***	−0.46 [−1.08, 0.10]	<0.001 ***	−0.20 [−0.69, 0.26]	0.018 *	0.21 [−0.14, 0.56]	0.014 *	② > ③ > ①
Extraversion	37.06	<0.001 **	0.25 [−0.22, 0.82]	<0.001 ***	0.08 [−0.36, 0.54]	<0.001 ***	−0.16 [−0.54, 0.20]	<0.001 ***	① > ③ > ②
Openness	1.97	0.373	−0.27 [−0.96, 0.34]		−0.07 [−0.66, 0.50]		0.21 [−0.13, 0.57]		
Agreeableness	8.58	0.012 *	0.67 [0.19, 1.24]	0.030 *	0.16 [−0.26, 0.63]	0.730	−0.46 [−0.84, −0.10]	0.014 *	①, ③ > ②
Conscientiousness	2.79	0.245	0.44 [−0.15, 1.15]		0.24 [−0.20, 0.72]		−0.12 [−0.46, 0.20]		

Note: Cluster 1: ①; Cluster 2: ②; Cluster 3: ③; d = Cohen’s d; 95% bootstrap confidence intervals (2000 iterations) are shown in brackets. Effect sizes are reported for all pairwise comparisons, regardless of statistical significance. All post hoc *p*-values are Bonferroni-corrected. * *p* < 0.05; ** *p* < 0.01; *** *p* < 0.001, and similarly for the following.

**Table 6:** Non-Parametric Tests and Post Hoc Tests of Psychological Symptoms.

	Non-Parametric Test		Post Hoc Test Comparisons						
			①–②		①–③		②–③		
	X^2^	*p*	d [95% CI]	*p*	d [95% CI]	*p*	d [95% CI]	*p*	
Somatization	9.10	0.110	−0.16 [−0.78, 0.45]				0.03 [−0.30, 0.39]		
Obsessive–Compulsive Symptoms	4.10	0.129	0.34 [−0.27, 1.00]		−0.12 [−0.65, 0.46]		−0.08 [−0.40, 0.24]		
Interpersonal Sensitivity	15.13	<0.001 ***	−0.18 [−0.79, 0.41]	<0.001 ***	0.22 [−0.28, 0.77]	0.086	0.23 [−0.14, 0.59]	<0.001 ***	② > ③, ①
Depression	6.89	0.032 *	0.04 [−0.61, 0.63]	0.041 *	0.01 [−0.62, 0.67]	0.613	0.11 [−0.25, 0.47]	0.038 *	② > ③, ①
Anxiety	11.89	0.003 **	0.22 [−0.40, 0.87]	0.013 *	0.15 [−0.44, 0.80]	0.778	0.09 [−0.24, 0.46]	0.002 **	② > ③, ①
Hostility	8.00	0.018 *	−0.16 [−0.76, 0.45]	0.018 *	0.30 [−0.30, 1.02]	0.380	−0.02 [−0.34, 0.33]	0.036 *	② > ③, ①
Phobia	8.73	0.013 *	−0.09 [−0.72, 0.49]	0.017 *	−0.16 [−0.67, 0.38]	0.469	0.06 [−0.31, 0.44]	0.020 *	② > ③, ①
Paranoid Ideation	7.98	0.019 *	0.08 [−0.72, 0.72]	0.021 *	−0.05 [−0.67, 0.61]	0.054	0.01 [−0.28, 0.34]	0.030 *	② > ③, ①
Psychosis	18.51	<0.001 ***	−0.03 [−0.61, 0.59]	0.020 *	0.08 [−0.52, 0.72]	1.000	0.25 [−0.09, 0.64]	<0.001 ***	② > ③, ①
Other	14.99	<0.001 ***	−0.13 [−0.72, 0.43]	0.003 **	0.22 [−0.32, 0.87]	0.466	0.24 [−0.10, 0.59]	<0.001 ***	② > ③, ①

Note: Cluster 1: ①; Cluster 2: ②; Cluster 3: ③. d = Cohen’s d; 95% bootstrap confidence intervals (2000 iterations) are shown in brackets. Effect sizes are reported for all pairwise comparisons, regardless of statistical significance. All post hoc *p*-values are Bonferroni-corrected. * *p* < 0.05; ** *p* < 0.01; *** *p* < 0.001.

**Table 7:** Distribution of Mental Health Risk Categories.

Risk Category	Risk Score	Number	Percentage
Low-Risk	0	84	55.6%
Medium-Risk	1	27	17.9%
High-Risk	2–3	40	26.5%
Total	0–3	151	100.0%

**Table 8:** Big Five Personality Traits under Different Mental Health Risk Statuses.

Personality Trait	Low-Risk Group (*n* = 84)	Medium–High-Risk Group (*n* = 67)	t/U	*p*	Cohen’s d
Neuroticism	2.26 ± 0.45	2.65 ± 0.52	−4.93	<0.001 **	0.81
Agreeableness	4.11 ± 0.41	3.89 ± 0.40	3.36	0.001 **	0.54
Conscientiousness	3.94 ± 0.57	3.69 ± 0.55	2.75	0.015 *	0.44
Openness	3.36 ± 0.54	3.31 ± 0.41	0.52	0.605	0.10
Extraversion	3.44 ± 0.45	3.20 ± 0.40	3.44	0.002 **	0.56

Note: Values are presented as M ± SD. Cohen’s d represents the standardised mean difference effect size (small = 0.2, medium = 0.5, large = 0.8). * *p* < 0.05; ** *p* < 0.01.

**Table 9:** Logistic coefficient of personality and mental health risk.

Predictor Variables	B	SE	Wald χ^2^	*p*-Value
Neuroticism	1.523	0.285	28.52	<0.001
Extraversion	−0.891	0.298	8.93	0.003
Conscientiousness	−0.562	0.243	5.35	0.021
Agreeableness	0.398	0.267	2.22	0.136
Openness	0.152	0.221	0.47	0.493
(Constant Term)	2.145	1.256	2.91	0.088

Note: B = unstandardized coefficient; SE = standard error; *N* = 151; model χ^2^(5) = 47.82, *p* < 0.001; Nagelkerke R^2^ = 0.368. VIF range: 1.26–1.91 (all < 2.0), indicating no multicollinearity.

**Table 10:** Sensitivity Analysis of the Risk Classification Algorithm Across Three Threshold Specifications.

Threshold	Criteria	N Medium/High (%)	AUC	95% CI	Sensitivity	Specificity	H-L *p*
Loose	any ≥ 1.5; mean ≥ 1.5; ≥ 3 dims ≥ 1.2	117 (77.5%)	0.683	[0.581, 0.785]	0.615	0.706	0.712
Standard	any ≥ 2.0; mean ≥ 2.0; ≥ 3 dims ≥ 1.5	67 (44.4%)	0.740	[0.660, 0.820]	0.701	0.690	0.778
Strict	any ≥ 2.5; mean ≥ 2.0; ≥ 3 dims ≥ 2.0	24 (15.9%)	0.785	[0.686, 0.884]	0.833	0.638	0.392

Note: AUC = area under the receiver operating characteristic curve; CI = confidence interval; H-L *p* = Hosmer–Lemeshow goodness-of-fit test *p*-value. The standard threshold set corresponds to the primary analysis reported in the main text. All models used the same five Big Five predictors with standardised scoring.

## Data Availability

The data involved in this study are available upon request from the corresponding author, but access is restricted due to privacy protezction. https://github.com/sheepissleeping/Specific-Associations-Between-Personality-Traits-Mental-Health-and-Emotional-Expression (accessed on 22 March 2026).

## Supplementary Materials

The following supporting information can be downloaded at: https://www.mdpi.com/article/10.3390/brainsci16040353/s1, Table S1. Sensitivity Analysis: Comparison of Kruskal-Wallis H Test Results Between Full Sample (n = 151) and Trimmed Sample Excluding Participants Aged 10–11 Years (n = 141). Table S2: The Big Five Inventory-2 (BFI-2). Table S3. Cluster Centroids Under Raw and Z-Score Standardized K-Means Solutions. Table S4. SCL-90 Inter-Subscale Cronbach’s α by Age Group. Table S5. Multiple Comparison Corrections: Bonferroni and Benjamini-Hochberg FDR Results for All 15 Outcome Variables. Table S6. Down-Sampling Sensitivity Analysis: Kruskal-Wallis Results Across 1000 Iterations (n = 17 per group).

## Abbreviations

The following abbreviations are used in this manuscript:

BFI-2	The Big Five Inventory–2
