# Supplementary material for: Clustering Analysis of Emotional Expression, Personality Traits, and Psychological Symptoms
Source: Brain Sci. 2026 Mar 25;16(4):353. doi: 10.3390/brainsci16040353 (PMC13113890; doi:10.3390/brainsci16040353)
Supplement: Supplementary file 1 [file brainsci-16-00353-s001.zip › brainsci-4183479-supplementary.pdf]

Table S1. Sensitivity Analysis: Comparison of Kruskal-Wallis H Test Results Between Full Sample (n = 151) and Trimmed Sample Excluding Participants Aged 10–11 Years (n = 141)

Variable	H	*p*	η^2	H	*p*	η^2	Conclusion
SCL-90 Subscales							
SOM	9.36	0.025	0.043	10.28	0.016	0.051	Consistent
OC	4.89	0.180	0.013	5.13	0.163	0.015	Consistent
IS	8.21	0.042	0.035	8.31	0.040	0.037	Consistent
DEP	8.44	0.038	0.037	9.25	0.026	0.044	Consistent
ANX	5.11	0.164	0.014	5.78	0.123	0.020	Consistent
HOS	6.26	0.100	0.022	6.07	0.108	0.022	Consistent
PHOB	8.00	0.046	0.034	7.74	0.052	0.033	Boundary effect
PAR	2.82	0.420	0.000	2.49	0.477	0.000	Consistent
PSY	5.51	0.138	0.017	5.64	0.131	0.019	Consistent
Big Five Personality Dimensions							
Neuroticism	17.09	<0.001	0.096	19.22	<0.001	0.114	Consistent
Extraversion	7.65	0.054	0.032	7.80	0.050	0.034	Consistent
Openness	7.65	0.054	0.032	6.68	0.083	0.026	Consistent
Agreeableness	9.85	0.020	0.047	11.90	0.008	0.063	Consistent
Conscientiousness	37.39	<0.001	0.234	42.17	<0.001	0.276	Consistent

Note. H = Kruskal-Wallis H statistic; η^2 = eta-squared effect size; SOM = Somatization; OC = Obsessive-Compulsive; IS = Interpersonal Sensitivity; DEP = Depression; ANX = Anxiety; HOS = Hostility; PHOB = Phobic Anxiety; PAR = Paranoid Ideation; PSY = Psychoticism. The trimmed sample excludes participants aged 10–11 years (n = 10). Highlighted row indicates the only variable with a difference in significance threshold between the two samples; the effect size (η^2) remained unchanged (0.034 vs. 0.033), reflecting a boundary p-value rather than a substantive change.

Table S2: The Big Five Inventory-2 (BFI-2)

Below are some descriptions of personal characteristics. Some may apply to you, while others may not. For example, do you agree with the statement: "I am a person who enjoys being around others"? Please fill in the corresponding number in the blank before each sentence to indicate how much you agree or disagree with the statement.

I am a person who...

1 - Strongly disagree | 2 - Disagree | 3 - Neutral | 4 - Agree | 5 - Strongly agree

1. ____ is outgoing and enjoys socializing.
2. ____ is soft-hearted and compassionate.
3. ____ lacks organization.
4. ____ remains calm and handles stress well.
5. ____ has little interest in art.
6. ____ is assertive and confident in expressing opinions.
7. ____ is humble and respectful toward others.
8. ____ is somewhat lazy.
9. ____ maintains a positive attitude even after setbacks.
10. ____ is interested in many different things.
11. ____ rarely feels excited or strongly desires something.
12. ____ frequently criticizes others.
13. ____ is trustworthy and reliable.
14. ____ experiences mood swings and emotional ups and downs.
15. ____ is creative and finds smart ways to do things.
16. ____ is relatively quiet.
17. ____ lacks empathy for others.

18. ____ is organized and plans ahead.
19. ____ is easily nervous.
20. ____ is fascinated by art, music, or literature.
21. ____ often takes the lead and behaves like a leader.
22. ____ frequently disagrees with others.
23. ____ finds it difficult to get started on tasks.
24. ____ feels secure and satisfied with themselves.
25. ____ dislikes intellectual or philosophical discussions.
26. ____ has less energy than others.
27. ____ is forgiving and tolerant.
28. ____ is sometimes irresponsible.
29. ____ is emotionally stable and rarely gets angry.
30. ____ has little creativity.
31. ____ is sometimes shy and introverted.
32. ____ is helpful and selfless toward others.
33. ____ keeps things neat and organized.
34. ____ often worries and is anxious about many things.
35. ____ values art and aesthetics.
36. ____ feels it is difficult to influence others.

37. ____ is sometimes rude to people.
38. ____ is efficient and follows through on tasks.
39. ____ often feels sad.
40. ____ has deep thoughts.
41. ____ is energetic.
42. ____ does not trust others and is suspicious of their intentions.
43. ____ is reliable and always trustworthy.
44. ____ is good at controlling emotions.
45. ____ lacks imagination.
46. ____ is talkative and enjoys conversation.
47. ____ is sometimes indifferent or cold toward others.
48. ____ is messy and disorganized.
49. ____ rarely feels anxious or afraid.
50. ____ finds poetry and drama boring.
51. ____ prefers letting others take the lead.
52. ____ is modest and courteous.
53. ____ is persistent and completes tasks.
54. ____ often feels down or depressed.
55. ____ is uninterested in abstract concepts and ideas.
56. ____ is enthusiastic.
57. ____ tends to see the best in people.
58. ____ sometimes behaves irresponsibly.
59. ____ is emotionally unstable and easily angered.
60. ____ is creative and comes up with new ideas.

Please check if you have filled in the corresponding number before each sentence.

Table S3. Cluster Centroids Under Raw and Z-Score Standardized K-Means Solutions

Method	Cluster	Anger	Disgust	Fear	Happy	Neutral	Sad	Surprise
Raw	Cluster 1 (n=17)	0.054	0.092	0.028	0.465*	0.130	0.148	0.083
	Cluster 2 (n=44)	0.083	0.083	0.053	0.135	0.236*	0.274*	0.136
	Cluster 3 (n=90)	0.154*	0.308*	0.061	0.085	0.136	0.172	0.082
Z-score	Cluster 1 (n=21)	0.059	0.100	0.027	0.427*	0.148	0.159	0.079
	Cluster 2 (n=43)	0.174*	0.294*	0.064	0.083	0.137	0.164	0.085
	Cluster 3 (n=87)	0.074	0.090	0.053	0.130	0.235*	0.280*	0.138

Note. Values represent mean emotion probability scores. Asterisk (*) indicates the dominant emotion for each cluster. Raw Cluster 1 corresponds to Z-score Cluster 1 (happiness-dominant); Raw Cluster 2 to Z-score Cluster 3 (neutral/sadness-dominant); Raw Cluster 3 to Z-score Cluster 2 (disgust/anger-dominant). Overall assignment agreement = 92.1% (139/151 participants).

Table S4. SCL-90 Inter-Subscale Cronbach's α by Age Group

Age Group	N	α	95% CI
Adolescents (10–18 years)	36	0.942	0.914–0.970
Young Adults (19–35 years)	17	0.948	0.914–0.982
Middle-aged (36–60 years)	59	0.933	0.908–0.957
Older Adults (≥60 years)	39	0.918	0.881–0.956

Note. α values are based on the nine SCL-90 subscale scores, reflecting inter-subscale internal consistency. 95% CIs estimated using the Fisher transformation method. α > 0.80 = good; α > 0.90 = excellent.

Table S5. Multiple Comparison Corrections: Bonferroni and Benjamini-Hochberg FDR Results for All 15 Outcome Variables

Variable	H	p	p (Bonferroni)	Significant	q (BH FDR)	Significant
SCL-90 Subscales						
SOM	9.364	0.025	0.348	No	0.084	No
O-C	4.891	0.180	1.000	No	0.194	No
I-S	8.211	0.042	0.586	No	0.084	No
DEP	8.444	0.038	0.528	No	0.084	No
ANX	5.108	0.164	1.000	No	0.191	No
HOS	6.264	0.100	1.000	No	0.139	No
PHOB	8.001	0.046	0.644	No	0.084	No
PAR	2.822	0.420	1.000	No	0.420	No
PSY	5.507	0.138	1.000	No	0.176	No
Big Five Dimensions						
Neuroticism	17.090	0.001	0.010	Yes	0.005	Yes
Agreeableness	9.846	0.020	0.279	No	0.084	No
Conscientiousness	37.387	<0.001	<0.001	Yes	<0.001	Yes
Openness	7.650	0.054	0.753	No	0.084	No
Extraversion	7.648	0.054	0.754	No	0.084	No

Note. H = Kruskal-Wallis H statistic; p = uncorrected p-value; p (Bonferroni) = Bonferroni-adjusted p-value from post hoc Dunn tests; q (BH FDR) = Benjamini-Hochberg false discovery rate adjusted q-value computed across all 15 outcome variables. SOM = Somatization; O-C = Obsessive-Compulsive; I-S = Interpersonal Sensitivity; DEP = Depression; ANX = Anxiety; HOS = Hostility; PHOB = Phobic Anxiety; PAR = Paranoid Ideation; PSY = Psychoticism.

Table S6. Down-Sampling Sensitivity Analysis: Kruskal-Wallis Results Across 1,000 Iterations (n = 17 per group)

Variable	Full H	Full p	Median p (Sensitivity)	Prop. Significant
Neuroticism	17.090	0.001	0.015	81.7%
Conscientiousness	37.387	<0.001	<0.001	100.0%
Agreeableness	9.846	0.020	0.120	28.0%
Extraversion	7.648	0.054	0.066	40.0%
Openness	7.650	0.054	0.249	14.5%

Note. Full H and Full p = results from the complete sample (N = 151). Median p = median p-value across 1,000 random iterations in which three larger age groups were sub-sampled to n = 17 to match the smallest group. Prop. Significant = proportion of iterations in which $p < 0.05$.
